# Cerebral Venous Sinus Thrombosis Following Scrub Typhus Infection: A Case Report and a Review of the Literature

**DOI:** 10.18103/mra.v10i10.3196

**Published:** 2022-10-31

**Authors:** Uttam Biswas, Ritwik Ghosh, Arghya Chakraborty, Shakilur Rahaman Mondal, Dipayan Roy, Arnab Bhattacharjee, Debanjan Roy, Julián Benito-León

**Affiliations:** 1 Department of General Medicine, Burdwan Medical College & Hospital, Burdwan, West Bengal, India;; 2 Department of Biochemistry, All India Institute of Medical Sciences (AIIMS), Jodhpur, Rajasthan, India;; 3 Indian Institute of Technology (IIT), Madras, Tamil Nadu, India;; 4 Department of Neurology, University Hospital “12 de Octubre”, Madrid, Spain;; 5 Centro de Investigación Biomédica en Red Sobre Enfermedades Neurodegenerativas (CIBERNED), Madrid, Spain;; 6 Department of Medicine, Complutense University, Madrid, Spain

**Keywords:** cerebral venous thrombosis, neuro-scrub, Orientia tsutsugamushi, scrub typhus

## Abstract

Neurological manifestations of scrub typhus, a re-emerging infectious disease of tropic/subtropics caused by *Orientia tsutsugamushi* infection, have been ever-evolving. Several central nervous system infections have been acknowledged for the development of cerebral venous sinus thrombosis (CVT). Nevertheless, CVT has been a rarely described addendum to the ever-evolving “neuro-scrub” spectrum. Proposed pathogenesis for the development of CVT is disseminated endotheliitis resulting in the triad of venous stasis (due to raised intracranial pressure), cerebral vasculopathy (endothelial damage), and capillary perivasculitis (endothelial damage and resultant hypercoagulable state generated by inflammatory mediators). We herein report a case of a previously healthy young female from the Indian subcontinent who was diagnosed with CVT, following scrub typhus. She responded well to conventional therapy with antibiotics and anticoagulants. CVT is amid the few completely reversible neurological catastrophes if diagnosed and treated early. Again, scrub typhus infection is treated with commonly available and extremely “affordable” antibiotics therapy. Hence, the authors propose that all cases of acute febrile illness with neurological manifestations from scrub-typhus endemic zones (like several parts of India) should be tested for the presence of *Orientia tsutsugamushi* infection and treated accordingly.

## Introduction:

The plethora of neurological manifestations of scrub typhus, a re-emerging tropical/subtropical infectious disease caused by *Orientia tsutsugamushi*, is ever-evolving.^1,2^ The invasion by the pathogen damages endothelial integrity and cytokines released by these inflamed endothelial cells lead to fluid permeation, platelet and polymorph aggregation, and monocyte proliferation, further resulting in focal obliterative angiitis and microthrombi formation in the vicinity of the leptomeninges, brain parenchyma, and venous sinuses.^1–6^ An eschar, which is often pathognomonic for scrub typhus infection found in intertriginous body portions as a bite mark of the vector (larval stage of trombiculid mite), is often absent and fails to provide an early clue to clinch the diagnosis.^7^ Several central nervous system (CNS) infections can result in the development of cerebral venous sinus thrombosis (CVT). However, the *Orientia tsutsugamushi* infection has rarely been associated with this neurological catastrophe.^6,8,9^

We herein report a case of a previously healthy young female from rural West Bengal (India) who presented a clinical picture of acute meningoencephalitis followed by CVT. After targeted evaluation, the etiology was an infection caused by a tick bite i.e., scrub typhus. She responded well to conservative therapy with antibiotics and anticoagulants. Risk factors for venous thromboses such as inherent hypercoagulable states, recent COVID-19 infection or vaccination,^10–12^ and other causes of acquired thrombophilia were excluded.^13^

## Case Report:

A 21 year-old female patient presented with a history of recent onset headache and fever since five days and fluctuating sensorium for the last day. The headache was severe, relentless, throbbing, and holocranial in nature (especially over the right occipital region), associated with vomiting and neck stiffness, and was not responding to pain-relief medications she took over-the-counter. Fever was abrupt in onset, of high grade, continuous, and associated with severe body aches, redness and pain in the eyes, and double vision. On examination, the patient was confused, febrile, and tachycardic. The neurological examination revealed a Glasgow coma scale score of 11/15, neck rigidity, and positive Kernig and Brudzinski signs. Extraocular movements revealed complete left lateral rectus palsy without any gaze paretic nystagmus. Motor, sensory, cerebellar, and autonomic symptoms were absent. Fundoscopic examination revealed grade 2 papilledema in both eyes.

**Figure 1:**
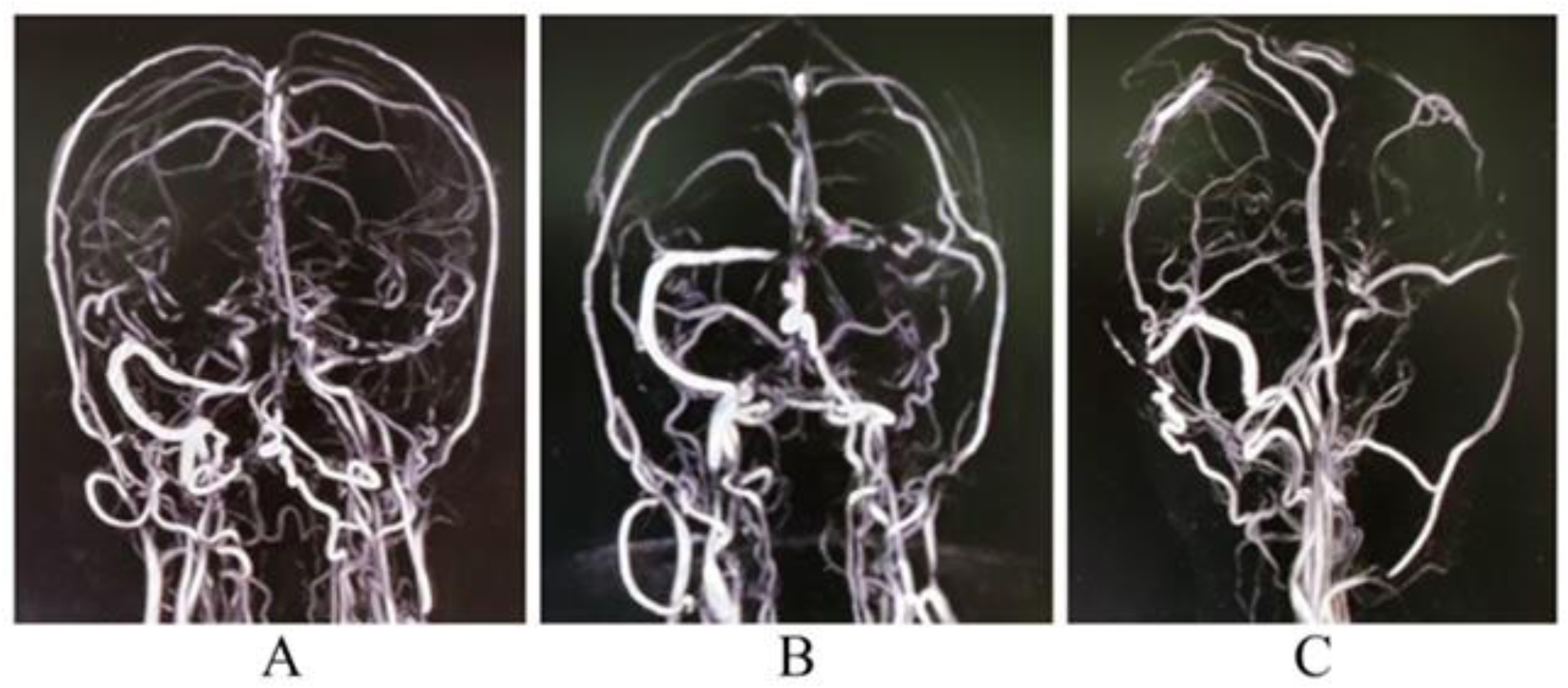
MR venography of the brain with gadolinium contrast (A, B, C) revealing thrombosis at left transverse sinus, straight sinus, posterior part of superior sagittal sinus, and at the torcular Herophilli with co-formation of prominent venous collaterals, suggestive of intraluminal obstruction.

Suspecting a clinical diagnosis of acute meningoencephalitis syndrome, she was empirically put on intravenous ceftriaxone, vancomycin, acyclovir, and other supportive therapies. Complete blood cell count revealed mild anemia (hemoglobin 9.8 g/dL), thrombocytopenia (90000/mm3), and raised erythrocyte sedimentation rate (66 mm in the first hour). Liver function tests revealed moderate transaminitis. Renal and thyroid functions, electrolytes, and blood glucose profiles were unremarkable. Tests for the malarial parasite, dengue, and typhoid serology were negative. The brain’s magnetic resonance imaging (MRI) with gadolinium contrast was ordered, which revealed no relevant abnormality. A cerebrospinal fluid (CSF) study revealed increased opening pressure, mononuclear pleocytosis (cell count 55/mm^3^, all lymphocytes), raised protein (83 mg/dL), and shallow glucose levels (13 mg/dL; corresponding capillary blood glucose was 113 mg/dL). CSF polymerase chain reaction (PCR) for relevant neuroviruses and *Mycobacterium tuberculosis* was negative. CSF and serum were tested for Scrub typhus IgM and PCR, which came positive. Acyclovir and vancomycin were stopped, and doxycycline was added. She became afebrile within four days of antimicrobial therapy. Glasgow coma scale score was 15/15 on day four, but the headache persisted. From day seven onwards, the lateral rectus palsy started to resolve, but the intensity of the headache was increasing. MR venography and brain angiography revealed thrombosis at the left transverse sinus, straight sinus, posterior part of superior sagittal sinus, and torcular Herophilli with co-formation of prominent venous collaterals (Figure 1). COVID-19 test was redone and was negative. Protein-C, protein-S, anti-thrombin-III, factor-V Leiden mutation, homocysteine, D-dimer, vitamin B12 levels, anti-phospholipid antibodies, lupus anticoagulants, anti-cardiolipin antibodies, antinuclear antibodies, and cryoglobulins were all negative. Serologies for hepatitis B, C, A, E, and HIV (1, 2) were also negative. She was immediately put on subcutaneous enoxaparin (80 mg/day) for five days, overlapped with 3 mg/day of oral warfarin with strict monitoring of prothrombin time and international normalized ratio. After five days of overlap therapy with enoxaparin and oral warfarin, her headache started improving, and by day 14 of hospital admission, she was symptomfree and discharged on warfarin 3 mg/day. No clinically demonstrable neurological deficits could be found on follow-up after eight weeks. However, repeat imaging showed only partial recanalization of the affected venous sinus lumen.

## Discussion:

The Asia-Pacific region has the highest prevalence of Rickettsial diseases. *Orientia tsutsugamushi* causes a life-threatening condition transmitted through the bite of the trombiculid mite.^1–3,14^ The parasite invades the CNS by hematogenous spread and resides in vascular endothelium and circulating phagocytes.^1–3,14^ The neuroinvasion results in host reaction and activates the cytokine and chemokine system, leading to parenchymal inflammation, perivasculitis, and infarction.^1–3,14^ Acute inflammation of the endothelial cells causes platelet aggregation, leukocytic infiltration, cytokine release, and thrombotic microangiopathy.^1–3,14^ Clinical features, therefore, can result from direct damage to an area causing altered functioning or may be immune-mediated.^1–3,14^

Severe scrub typhus is associated with interstitial pneumonia, renal failure, gastrointestinal bleeding, and CNS manifestations like meningoencephalitis (most common), cerebral infarction, transverse myelitis, movement disorders, and cranial neuropathy.^1–5,14^ Regardless, CVT is a rare manifestation. So far, only three cases of CVT in scrub typhus have been reported, all from India (Table 1). The complement system and the coagulation cascade are closely interlinked systems. The hyper-immune response in COVID-19 can lead to widespread thrombosis through complement activation, platelet activation and the coagulation cascade.^10,15^ We excluded COVID-19 and other possible factors for the occurrence of CVT in this case through extensive investigations. A hypercoagulable state, manifesting due to endotheliitis and vasculopathy and increased intracranial pressure, probably gave rise to venous stasis and venous thrombosis.

## Conclusions:

In closing, head/neck and/or systemic infections and CVT have an age-old nexus that has been recently gained quite an attention with advent of COVID-19.^10,11^ Scrub typhus can have varied and peculiar neurological manifestations.^1–5,14^ Amid those, CVT is an infrequent complication that is potentially treatable with prompt diagnosis. We propose that all cases of acute undifferentiated febrile illness with neurological manifestations from scrub-typhus endemic zones (like several parts of India) should be tested for the presence of *Orientia tsutsugamushi* infection (even in the absence of a pathognomonic “eschar”) and treated accordingly.

## Figures and Tables

**Table 1:** Reported cases of cerebral venous sinus thrombosis with scrub typhus infection

Authors	Patient	Presentation	CSF study	Neuroimaging	Treatment	Outcome
Das et al. 2021 ^[6]^	Female/32 years	High remittent fever, holocranial headache, vomiting, and confusion	Protein raised (120 mg/dL); normal glucose and adenosine deaminase; lymphocytic pleocytosis (30 cells/mm^3^)	MR venography: thrombosis of the terminal portion of the superior sagittal sinus, straight sinus, right sigmoid, and transverse sinus; brain CT scan showed diffuse swelling with no focal lesion; brain MRI showed no stroke or demyelinating lesion	Doxycycline (IV first two days, then 100 mg twice daily next ten days); mannitol infusion 100 mL three times a day until detection of venous thrombosis; enoxaparin 60 mg/d for three days, then changed to warfarin	Recovery
Jena et al. 2014 ^[8]^	Male/48 years	Fever, headache, vomiting, right-sided focal seizure, and altered sensorium	Not reported	MR venography: thrombosis of the anterior portion of the superior sagittal sinus with hemorrhagic venous infarct in the left frontal lobe; brain MRI brain showed an acute hemorrhagic venous infarct and swelling in the left high frontal region	Doxycycline, azithromycin, unfractionated heparin, and left frontotemporoparietal decompressive craniectomy	Partial recovery
Sardana & Shringi 2018 ^[9]^	Male/17 years	Throbbing headache, high-grade fever, and periorbital pain	Not reported	MR venography: acute thrombus in the superior sagittal sinus; brain MRI showed white matter edema in the bilateral high frontoparietal region	Doxycycline 100 mg twice daily, azithromycin 500 mg once a day, subcutaneous low-molecular-weight heparin, and acenocoumarol.	Recovery
Ghosh et al. 2022 (current study)	Female/21 years	Throbbing headache, fever, and altered sensorium	Protein raised (83 mg/dL), very low glucose (13 mg/dL) and lymphocytic pleocytosis (55 cells/mm^3^)	MR venography: thrombosis of the left transverse sinus, straight sinus, posterior part of superior sagittal sinus, and torcular Herophilli	Doxycycline 200 mg/day, subcutaneous enoxaparin 80 mg/day and oral warfarin 3 mg/day	Recovery
